# Changes in Prices After an Excise Tax to Sweetened Sugar Beverages Was Implemented in Mexico: Evidence from Urban Areas

**DOI:** 10.1371/journal.pone.0144408

**Published:** 2015-12-14

**Authors:** M. Arantxa Colchero, Juan Carlos Salgado, Mishel Unar-Munguía, Mariana Molina, Shuwen Ng, Juan Angel Rivera-Dommarco

**Affiliations:** 1 Center for Health Systems Research (CISS), Instituto Nacional de Salud Pública (INSP), Cuernavaca, Morelos, México; 2 Nutrition and Health Research Center (CINyS), Instituto Nacional de Salud Pública (INSP), Cuernavaca, Morelos, México; 3 Department of Nutrition and Carolina Population Center, University of North Carolina at Chapel Hill (UNC), Chapel Hill, North Carolina, United States of America; University of Washington, UNITED STATES

## Abstract

In 2014 an excise tax to non-alcoholic sweetened beverages (SSB) was implemented in Mexico. The objective of this paper is to study whether and to what degree these taxes passed-through onto SSB prices in urban areas overall and by region, type of beverage and package size. Prices were obtained from the National Institute of Statistics and Geography from 2011 to 2014. We applied a pre-post quasi-experimental approach using fixed effects models. In sensitivity analysis we applied other model specifications to test the robustness of the findings and we also present weighted estimations based on household purchases. The dependent variables are real prices of a specific beverage category; the main independent variables are dummies for each month of 2014, and the models adjust for time trends and seasonality. Results suggest that the SSB tax passed along to consumers for all SSBs and we found overshifting for the carbonated SSBs. A greater effect is seen among the small package sizes, and we see heterogeneous effects by region. Estimating the effect of the tax on prices is important to understand the potential effect on consumption.

## Introduction

The prevalence of overweight and obesity in Mexico has reached 73% of the adult women population, 69% of men and more than 30% of children and adolescents [1,2]. Mexico ranks second on obesity and first on diabetes prevalence of all countries members of the Organization for Economic Cooperation and Development [3,4]. Although obesity and chronic diseases are caused by multiple factors, evidence shows that consumption of sweetened sugar beverages (SSB) is a risk factor for obesity, type two diabetes and heart disease [5–9]. In 2011, Mexico had the largest per capita consumption of soft drinks worldwide estimated at 163 liters per capita per year [10]. Recent evidence shows that 71% of the consumption of added sugar in Mexico comes from SSB and at least 66% of the population exceeds the WHO recommendation by consuming more than 10% of their calories from added sugars [11].

In this context, the Mexican Congress implemented a tax initiative as a public policy aimed at limiting Mexico’s obesity epidemic [12]. On January 1, 2014 the government began instituting a one peso (0.008 USD) per liter excise tax on any non-alcoholic beverage (powder, concentrates or ready to drink) with added sugar with the exception of medical beverage products. The tax will be adjusted for inflation when inflation exceeds 10% which in general is approximately every two year. An excise tax is defined as a fixed amount per unit of product, which in the Mexican regulation is a one peso per liter and it has to be paid by the producer. A one peso per liter represents approximately a 10% increase in price at the time when the tax was passed (September 2013).

The theoretical implications of tax incidence (the effects of the tax on prices) have been described by economists as being dependent on the structure of the market and price elasticities. In markets with perfect competition, prices can increase by the complete amount of the tax if the long-term supply curve is horizontal–a very small change in price leads to a large change in quantity supplied, i.e., the elasticity of the supplier is close to infinity- and less than the amount of the tax if the curve is upward sloping [13]. In markets with imperfect competition, prices are usually above their marginal costs and taxes can passed to consumer prices more than the amount of the tax (overshifting) or less than the amount of the tax (undershifting) depending on the elasticity and cost function [13–16]. Young describes two potential mechanisms to explain overshifting in non-competitive markets [17]. If a firm faces a downward supply curve for a commodity, average costs are lowered due to economies of scale or other factors and if the price elasticity of the demand is high, the price will increase. But the taxes can also reduce the quantity demanded increasing the average total costs leading to an increase in prices that can be higher than the amount of the taxes. Companies in this scenario can decide to increase prices to compensate for revenue losses [18]. If the cost and demand functions are linear—a linear function of demand implies a non-constant elasticity that may generates strategies of segmented markets- or weakly convex demand the tax won’t pass to the consumers [18,19]. In monopolistic or oligopoly markets, firms can increase prices when consumer’s “loyalty” is high even if the market offers similar products and lower prices [16].

Empirically, Besley’s study on the effect of taxes on prices of different commodities in the United States (food and non-food items) confirms the economic theory that different shifting to price patterns is possible because the structure of the market varies by product[15]. A study on tobacco taxes show evidence that under oligolopy markets, there has been overshifting[20]. Similarly, Kernel shows overshifting in changes in prices after the taxes to alcoholic beverages increased in Alaska in 2002 [21].

Whether this holds in the case of SSBs is unclear as only a few countries have implemented SSB taxes, and there is little evidence on the effect on prices among these, except for two working papers conducted in France and in Denmark, and two published studies in the US and France [18,22–24]. In France, starting in January 2012, the government implemented a tax to sodas, fruit drinks and flavored waters of 7.16 euros per hectoliter (equivalent to about 6% of the average soda price). Results from the study show that after six months, prices rose more than the tax to prices for sodas and it was incomplete for fruit drinks and flavored water [22]. In Denmark, the paper reports results from three periods of tax increases on soft drinks: in 1998 the tax increase from 0.80 to 1 Danish Krone per liter (0.14 to 0.18 USD), to 1.6 in 2001 (0.28 USD) and a decrease in 2003 to 1.15 (0.20 USD). The authors found that the tax fully passed though prices for the tax cut in 2003 and an overshifting (exceeding full shifting) for the two periods of tax increases [18]. A paper published in the US estimated the effect of the existing sales taxes on soft drinks that on average represent 5% of price [23]. The results show overshifting but the effect was not significant when adding lags of the tax in the regression models. Another study on the effect of different taxes to soft drinks implemented in France on prices finds in a simulation that an excise tax to SSB shows overshifting [24].

In Mexico, all carbonated beverages (regular and diet beverages) account for more than 85% of the beverages sales (excluding still plain water): authors estimations using the Monthly Surveys of the Manufacturing Industry show that in 2013 sales of carbonated beverages reached $594.4 million dollars compared to $94.3 million dollars for non-carbonated beverages (juices and flavored water) [25]. The beverage market in the country is an oligopoly as the principal producer of carbonated beverages account for 70% of the sales, with the second largest firm accounting for 15% of sales [26]. Non-carbonated beverages are on average more expensive and more price elastic compared to carbonated sugar beverages [27]. In this context of imperfect competition with differences in both average prices and price elasticities of demand, we hypothesize that the prices of carbonated sugar sweetened beverages will change more than the amount of the tax (1 peso per liter) and less than the amount of the tax for non-carbonated sugar sweetened beverages.

The objective of this paper is to present the analysis of the effect of the Mexican SSB tax on prices in urban areas using price data from 46 urban areas from 2011 to 2014 and to see if there is heterogeneity by region, type of beverage and package size. Estimating the effect of the tax on prices to see if the tax passed along to consumers is a relevant piece of information to further estimate and understand the potential effect on consumption or purchases because the effect of the tax on consumption may be attenuated if the tax fails to pass along to consumers. Taxes may not pass along onto consumer prices when the producers absorbs the costs, which may include strategies to increase prices of other products to compensate, price discrimination (different prices for the same product by region or retailer), and other marketing strategies such as promotions.

## Materials and Methods

### Price Data

Prices were obtained from the National Institute of Statistics and Geography (INEGI), the entity currently responsible for collecting price data to estimate the Consumer Price Index in Mexico[28]. The Consumer Price Index measures average price changes of a basket of goods and services representative of all purchases from urban households over time. INEGI uses household expenditure information from the National Household Income Surveys to identify all good and services purchased by urban dwellers that are then classified by INEGI into 283 types of products[29]. Prices are collected in 46 cities distributed across the country, ensuring available information for each the 32 states of Mexico. These cities have a population above 20,000 habitants including the 10 most populated urban zones in the country [29]. In each city, prices are obtained from a non-probabilistic sample of 16,000 points of sales (e.g. stores, vendors). Food and beverage prices are collected weekly during the year from the different points-of-sales. All prices include taxes to reflect consumer prices. Prices were deflated using the consumer price index for 2010[28].

We retrieved average monthly price data from January 2011 to December 2014 for the all ready to drink beverages. Monthly price data is the average weekly data collected by INEGI. For each beverage item in the data set we also extracted package size information, in order to determine if there was also heterogeneity in passing on the beverages to larger compared to smaller package versions of similar products.

### Purchase Data

Although enumerators from INEGI are requested to collect prices data on the items more frequently consumed, the data at the food and beverage level are not weighted based on consumption or purchases. To present weighted and unweighted estimations, price data from INEGI was weighted using Nielsen purchase data from January 2012 through March 2014, from The Nielsen Company-Mexico’s Consumer Panel Services. We derive volume distributions of beverages purchases by beverage category (all sweetened sugar beverages, carbonated sweetened and non-carbonated sweetened beverages), month, year and geographic location. For 2011, we imputed the volume distributions estimated for 2012 by month and geographic location. Volume distributions that varied over time and geographic location were used as survey weights in the analysis.

### Descriptive Analyses

We first describe real average monthly prices (base price at 2010) of taxed and untaxed beverages to compare monthly trends over time (2011–2014). Under taxed beverages we disaggregate by carbonated SSBs such as soft drinks, non-carbonated SSBs such as juices and flavored waters. For the untaxed beverages we present trends in prices for sparkling water, water, juices and flavored water and diet soft drinks.

### Empirical Estimation

Since the tax was implemented nationally, it was not possible to have a true experimental design to study the effect of the SSB tax. Therefore, we applied a pre-post quasi-experimental approach. We used fixed effects models to control for all variables that do not change over time. We also included variables that change with time and can explain changes in the demand and prices of beverages such as annual projected population[30] and annual gross domestic product[31].

We applied the model to all SSBs that are subject to the tax and we also stratified the models by two beverage categories given the differences described in terms of their prices and price elasticities: 1) carbonated sweetened beverages (CSB) that includes non-diet soft drinks and 2) non-carbonated sweetened beverages (NCSB) including flavored water, juices and nectars. The fixed effects model is laid out as follows:

$$P_{itj}\;=\;{\delta Y+\beta }_{y}Y+\beta_{m}M+\beta_{m2}M2+\beta_{s}S+\beta_{p}Do+\beta_{g}G+\alpha_{i}+u_{itj}$$

The dependent variable *P* is the real price per liter of a specific beverage *i* at month *t*, year *j*, *Y* is a vector of dummies for each calendar year (leaving 2013 as the reference), *M* is a count variable for the entire month/year period 2011–2014, *M2* is the variable month squared to test for non-linear associations, *S* denotes the season that takes the value 1 during the period of higher prices (April to September), 0 otherwise, D is total annual population projected (in millions) in the country and *G* is the annual gross domestic product in the previous year in millions of real pesos, *α* are time invariant unobservable factors associated with *P* and *u* is the error term.

To see how the taxes passed over time, we included a model where we adjust for each month of 2014 and compares prices with 2013.

We explored the heterogeneity of the effect of the tax on SSB prices by region and the two beverage categories (CSB and NCSB) and we also ran the fixed effects models by package size to see if there were differences in changes in price after the tax was implemented.

To test the robustness of the results, we applied two other model specifications as sensitivity analyses. First, we used an Arellano-Bond Dynamic Panel Estimator that includes lag of prices as instrument variables for endogenous regressors and that addresses the potential autocorrelation[32]. We also applied interrupted time-series analyses (ITSA) that have been used to estimate in non-experimental designs the impact of tobacco taxes[33]. As in time series, for the ITSA price data are collapse by month to run the estimations. All models adjust for the same variables as in the fixed effects regression for comparison. Results from all models are presented showing unweighted and weighted estimations using Nielsen purchase data as described above. For fixed effects, we used the command–*areg-* in Stata (linear regression with a large dummy-variable set) that allows using time variant weights. All empirical analyses were run in Stata 13[34].

## Results

Table 1 shows volume distributions by beverage category estimated using Nielsen household purchase data from January 2012 to March 2014. CSB represent about 85% of the purchases and NCSB represent 15% of purchases. Within CSBs, on average 87% are purchases of beverages with package sizes greater than 600ml; 6% for 600ml and 6.5% for the smallest package sizes.

**Table 1:** Average volume distribution by beverage category using Nielsen purchase data.

Beverage category	% Volume distribution [standard deviation]		
	All sugar sweetened beverages	Carbonated sweetened beverages	Non carbonated sweetened beverages
Carbonated sweetened beverages			
< 600ml	5.6 [4.8]	6.6 [5.6]	**-**
600ml	4.9 [2.9]	5.9 [3.6]	**-**
>600ml	74.5 [7.8]	87.0 [7.0]	**-**
Total	**84.0**	**100**	**-**
Non carbonated sweetened beverages			
Flavored water	2.7 [1.6]	**-**	19.3 [11.7]
Juices < 1liter	3.8 [1.6]	**-**	26.2 [9.3]
Juices 1 liter	2.4 [1.4]	**-**	15.8 [7.5]
Juices > 1 liter	6.5 [3.7]	**-**	38.2 [12.7]
Total	**16.0**	**-**	**100**

*Source*: authors’ own analyses and calculations based on price data from INEGI and The Nielsen Company through its Mexico Consumer Panel Service (CPS) for the food and beverage categories for January 2012 –December 2014. Copyright 2014, The Nielsen Company. The Nielsen Company has no responsibility in the results reported.

**Fig 1:**
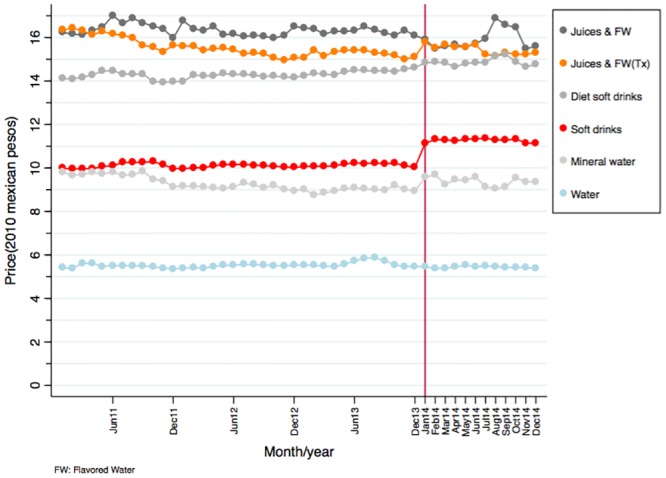
Average price per liter by type of beverage (unadjusted).

Fig 1 shows monthly unweighted average real prices per liter between January 2011 and December 2014 for taxed and untaxed beverages. The graph shows a smooth and stable trend in prices for soft drinks (taxed CSB) and an increase of about 1 peso per liter beginning in January 2014 that continues until the end of the year. In contrast, taxed juices and flavored waters varied more prior to the tax and the price increase in January 2014 was smaller and varied during the year. For the untaxed beverages, water displays a very stable trend over time with no significant changes after 2014. Prices of diet soft drinks are on average more expensive than regular soft drinks and their prices increased over time since 2011 but after the tax the difference became smaller. Mineral water prices slightly increase after 2014.

Table 2 shows results from the fixed effects models for all SSB to see how the tax passed through prices over 2014 by region. Overall we see that prices increases close to one peso per liter since January 2014 and remained over the year around that level except for November and December that were lower. We see that there is heterogeneity on the effect of the SSB tax on prices by region, we see overshifting in Mexico City, Central North, North Border and the Northwest but undershifting for the rest, where it is particularly low in the South.

**Table 2:** Fixed-effects estimating the change in SSB prices (pesos per liter) after the excise tax started by region.

Variable	All	Mexico City	Central North	Central South	North Border	Northeast	Northwest	South
January	0.95 **	1.00 **	1.32**	1.14**	0.78**	0.65**	0.82**	0.68**
February	1.00 **	0.95**	1.24**	1.04**	1.05**	0.86**	1.17**	0.64**
March	1.05 **	1.00 **	1.27**	1.10**	1.08**	0.94**	1.17**	0.71**
April	0.93**	1.00**	1.23**	0.83**	1.04**	0.63**	1.09**	0.75**
May	1.02**	1.03**	1.32**	0.93**	1.19**	0.61**	1.15**	0.96**
June	1.04**	1.11**	1.42**	0.86**	1.26**	0.63**	1.36**	0.87**
July	0.98**	0.98**	1.41**	0.93**	1.05**	0.48*	1.36**	0.70**
August	0.98**	1.12**	1.40**	0.89**	1.16**	0.42+	1.23**	0.71**
September	0.98**	1.06**	1.43**	0.88**	1.16**	0.34	1.17**	0.92**
October	1.03**	1.16 **	1.50**	1.05**	1.14**	0.34	1.04**	0.97**
November	0.91**	1.24**	1.35**	0.83**	1.04**	0.25	1.12**	0.79**
December	0.90**	1.19 **	1.30**	0.85**	1.06**	0.19	1.09*	0.90**
2012	0.01	-0.04	-0.08	-0.19+	0.14	0.15+	0.40*	-0.07
2011	-0.06	-0.19	-0.15	-0.34+	0.21	0.21	0.11	-0.12
Month	0.00	-0.01	0.02	0.01	-0.02	-0.02	0.01	-0.00
Month squared	-0.00	0.00	0.00 +	-0.00	0.00	0.00	-0.00	-0.00
High-season	0.09**	0.13	0.06	0.07**	0.05	0.20**	0.04	0.08
Lagged GDP^a^	-0.05	0.02	-0.13	-0.17	-0.04	0.16	-0.14	0.05
Population ^b^	0.03	-0.05	-0.05	-0.05	0.26	0.09	0.06	0.04
N	26,307	1,717	5,467	5,456	3,386	4,689	2,061	3,531

*Source*: authors’ own analyses and calculations based on price data from INEGI (2011–2014).

^a^ National Annual Gross Domestic Product in millions of real pesos (2010 base), lagged = previous year,

^b^ Annual national projected population per million of inhabitants,

** p < .01,

* p < .05,

^+^p<0.1

Table 3 shows the results of the fixed effects stratified by package size for CSB and NCSB. For both groups, price changes were higher among the beverages with smaller package sizes. Additionally the price increase was greater for CSBs (overshifting) but much lower than one peso per liter (undershifting) for NCSBs.

**Table 3:** Fixed-effect models estimating the change in prices for carbonated sugar sweetened beverages (CSB) and non-carbonated sweetened beverages (NCSB) after the excise SSB tax started stratified by package size.

Package size	Price change	Observations
**CSD**		
< 600ml	1.50 [0.12]**	2,785
600ml	1.23 [0.04]**	6,788
600-1liter	1.13 [0.10]*	800
>1liter	1.08 [0.04]**	7,642
**NCSD**		
< 1liter	0.61 [0.12]**	3,770
1 liter	0.75 [0.11]**	3,696
> 1liter	0.36 [0.14]**	826

*Source*: authors’ own analyses and calculations based on price data from INEGI (2011–2014). All models are adjusted for calendar year (2013 reference), month as a count, month squared, high season, national annual gross domestic product in the previous year and annual population

** p < .01,

* p < .05;. standard error in brackets.

The sensitivity analysis to various empirical specifications and comparisons between weighted and unweighted models is presented in Table 4. The unweighted and weighted estimations were slightly different in all estimations. In general, the regressions using Arellano Bond Panel Estimations were lower compared to fixed effects and interrupted time series analysis however the overall results are similar.

**Table 4:** Different model specifications estimating the change in prices after the excise SSB tax started by beverage type (unweighted and weighted).

Model specification	Sugar Sweetened Beverages	Carbonated Sweetened Beverages	Non-carbonated Sweetened Beverages
**Fixed effects** +	n = 26,307	n = 18,089	n = 8,292
Unweighted	1.03**	1.20**	0.66**
Weighted	1.08**	1.10**	0.74**
**Arellano Bond Dynamic Panel Estimation**	n = 24,548	n = 16,892	n = 7,686
Unweighted	1.00**	1.08**	0.70**
Weighted	0.93**	0.96**	0.70**
Interrupted time series analysis*	n = 48	n = 48	n = 48
Unweighted	1.12**	1.15**	0.53**
Weighted	0.95**	1.14**	0.57**

*Source*: authors’ own analyses and calculations based on price data from INEGI 2011–2014 and volume distributions for the weighted regressions based on data from The Nielsen Company through its Mexico Consumer Panel Service (CPS) for the food and beverage categories for January 2012 –December 2014. Copyright 2014, The Nielsen Company. The Nielsen Company has no responsibility in the results reported.

*Note*: All models are adjusted for calendar year (2013 reference national annual gross domestic product in the previous year and annual population. Weighted using Nielsen volume distribution. The number of observations varies by model specification, the Arellano Bond Dynamic Panel Estimation has a lower sample size because it uses lags,

^+^ Fixed effects in Stata using areg, absorb.

* the interrupted time series analysis collapses the data into 48 months.

** significant at 1%

## Conclusions

We estimated the effect of the SSB tax on prices in urban areas using publicly available price data applying fixed effects models unweighted and weighted according to household purchase distributions based on Nielsen purchase data. Our estimations show that prices passed along to consumers for all SSB as price changes in 2014 compared to 2013 were close to one peso per liter. We see overshifting for the CSB category as changes in prices were higher than one peso per liter. In contrast, changes in price were much lower than one peso for the NCSB. The results were also robust to other model specification: Arellano Bond Dynamic Panel Estimation and Interrupted Time Series Analysis. Overall, changes in SSB prices in urban areas from the different model specifications (weighted and unweighted) are in the range of 0.95 to 1.12 pesos per liter; price changes for CSB are between 0.96 and 1.20 and between 0.53 to 0.74 pesos per liter for NCSB.

Our findings are in accordance with the economic theory that predicts that under an oligopoly market, taxes can pass through prices more or less than the amount of the tax depending on the firm’s costs, market structure and the demand. For CSBs in Mexico, where 85% of the sales are concentrated between two firms, overshifting was expected. In contrast, we see undershifting of the tax for NCSB, which has a lower market share compared to soft drinks, and higher prices and price elasticity of the demand [27], despite also being produced by only a few companies.

We found differences in price changes by region and package size. Increases in prices were higher for the smallest package sizes both for CSB and NCSB, which may reflect producer’s strategies to avoid discouraging the consumption of large package beverages that are more penalized by the excise tax. These findings also highlight the importance of monitoring industry response to the tax, as the differential pass-through by package size could result in promoting purchases of larger package sizes and hence be counter-productive against the objective of the tax.

We found an incomplete pass-through of the tax on prices in some regions, particularly in the South with a price increase of 7% between 2013 and 2014, one of the regions with the lowest SSB prices in the country.

For CSB, weighted coefficients were slightly lower compared to unweighted results because according to Nielsen data the majority of purchases are from largest package sizes (>600ml) and our stratified analysis showed that changes in prices were lower for these sizes. It is possible that the Nielsen data may underestimate the percent of smaller package sizes due to purchases and consumption “on-the-go”. Nonetheless, our conclusions of an overshifting for CSB remain.

We acknowledge that in the absence of an experimental design, our estimations rely on a before and after approach. However, in the models, we used data from 2011 to capture previous trends in prices, we adjusted for time variant factors that could be associated with changes in prices and we applied different model specifications to show the robustness of the results. In addition, descriptive analyses show that prices of untaxed beverages did not changed after the SSB tax was implemented (except for sparkling water). This is the case of still plain water and diet soft drinks whose prices were increasing long before the tax. We also recognize that the study did not take into consideration promotions of SSB in stores because the INEGI data exclude prices of commodities that have promotions. Finally, for confidentiality reasons, the price data are not displayed by stores so we could not analyze the heterogeneity on effect of the tax on prices by store.

Price changes for CSB represent about 11% increase in price based on the average price in 2013 of 10.1 pesos per liter and the range of price increase in 2014 compared to 2013 estimated from the models. In contrast, given that the average price for NCSB is higher than the CSB prices (14.7 pesos per liter in 2013), the range of increase between 2013 and 2014 represented only a 3% increase.

Findings from this research are similar to a study in France that found overshifting in soft drinks and incomplete pass for juices and flavored water, although in Mexico the tax passed through prices since the first month the regulation was implemented in contrast with France where the complete shifting was observed after six months[22]. Our results also confirm findings from another study in France that shows overshifting for excise taxes to soft drinks in contrast to a sale tax[24].

As of our knowledge, this if the first paper that analyses the effect of the excise tax to SSB that was implemented since January 2014 using price data collected regularly by INEGI to estimate Consumer Price Index, as well as along with household purchases data. As the new regulation aims at reducing SSB consumption, evidence of an overshifting is the first condition to expect a decrease in consumption or purchases. The later would eventually depend on the price elasticity of the demand for SSB as well as market strategies from producers to promote the consumption of SSB despite the price increase such as promotions and other factors determining beverage consumption in the country.
